# A pharmacological inhibitor of NLRP3 inflammasome prevents non-alcoholic fatty liver disease in a mouse model induced by high fat diet

**DOI:** 10.1038/srep24399

**Published:** 2016-04-14

**Authors:** Gabsik Yang, Hye Eun Lee, Joo Young Lee

**Affiliations:** 1 Integrated Research Institute of Pharmaceutical Sciences, College of Pharmacy, The Catholic University of Korea, Bucheon, Republic of Korea, 420-743, South Korea

## Abstract

The activation of NOD-like receptor family pyrin domain containing 3 (NLRP3) inflammasome is closely associated with the development and progression of non-alcoholic fatty liver disease (NAFLD) induced by a high-fat diet. Therefore, we investigated whether oral administration of sulforaphane (SFN) prevented high-fat diet-induced NAFLD in mice by regulation of the NLRP3 inflammasome in the liver. Daily oral administrations of SFN reduced hepatic steatosis scores, serum ALT and AST levels, and hepatic levels of cholesterol, triglycerides, and free fatty acids in mice fed a high-fat diet. These were correlated with the suppression of NLRP3 inflammasome activation in the liver by SFN as evidenced by decrease in mRNA levels of ASC and caspase-1, caspase-1 enzyme activity, and IL-1β levels. SFN inhibited saturated fatty acid-induced activation of the NLRP3 inflammasome in primary mouse hepatocytes, accompanied by the restoration of mitochondrial dysfunction. The suppression of NLRP3 inflammasome by SFN was mediated by the regulation of AMP-activated protein kinase-autophagy axis. Our findings demonstrated that the suppression of NLRP3 inflammasome activation by an orally available small molecule inhibitor leads to the alleviation of the hepatic steatosis symptoms associated with NAFLD induced by a high-fat diet.

Non-alcoholic fatty liver disease (NAFLD) is a broad-spectrum liver disease with symptoms ranging from simple steatosis to liver failure. NAFLD is a condition that is not caused by alcohol and is defined by excessive fat accumulation involving greater than 5 to 10% of the liver (steatosis). A subgroup of NAFLD patients develop the more severe form of NAFLD, non-alcoholic steatohepatitis (NASH), exhibiting liver cell injury and inflammation in addition to excessive fat (steatohepatitis)^1^,^2^.

It is well established that interleukin-1β (IL-1β) promotes the recruitment of inflammatory cells to the liver and activates hepatic stellate cells, contributing to the development and progress of fibrosis^3^. IL-1β induces triglyceride accumulation in hepatocytes^3^,^4^ and triggers hepatocytes death together with TNF-α^5^. IL-1β secretion is mainly dependent on activation of the inflammasome complex^6^. Inflammasomes are multiprotein complexes that assemble upon danger signals and initiate the release of IL-1βand IL-18 via caspase-1 activation^7^. Emerging evidence supports a central role of the NOD-like receptor family pyrin domain containing 3 (NLRP3) inflammasome, comprising NACHT, LRR NLRP3, apoptosis-associated speck-like CARD-domain protein (ASC), and caspase-1, in the pathogenesis of various liver diseases including NAFLD, liver fibrosis, ischemia/reperfusion injury, and drug-, pathogen-, or endotoxin-mediated pathology^8^,^9^,^10^,^11^. Saturated fatty acids induce activation of the NLRP3 inflammasome, suggesting that the saturated fatty acids abundant in a high-fat diet are the key players in the inflammasome regulation^12^,^13^. Given that elevated concentrations of free fatty acids in the plasma are associated with the development and promotion of NAFLD and NASH^14^, sustained NLRP3 activation by free fatty acid could provide a critical hit to aggravate the progression towards a more severe liver phenotype along with the preceding hit of obesogenic stress during NAFLD development. The central role of inflammasome activation in the pathogenesis of liver disease makes its inhibition an attractive target for the treatment of these disorders; in particular as only limited number of treatments is available for the liver disease^7^.

We attempted to find orally administrable small-molecule inhibitors that target the NLRP3 inflammasome for possible preventive application in the management of NAFLD. In this study, we found that sulforaphane (1-isothiocyanato-4-methylsulfinylbutane; SFN) suppressed the activation of the NLRP3 inflammasome activation induced by saturated fatty acid in hepatocytes. SFN is an organosulfur compound that exhibits potent anti-inflammatory activity and was recently reported to attenuate high fat diet-induced visceral adiposity, adipocyte hypertrophy, lipid accumulation^15^, and alcohol-induced steatosis^16^. However, whether SFN prevents NAFLD has not been investigated, and the underlying mechanism by which SFN improves hepatic steatosis is poorly understood. Therefore, we investigated whether SFN prevented high fat diet-induced NAFLD in mice and whether this prevention was mediated by regulation of the NLRP3 inflammasome in the liver.

## Results

### Oral administration of sulforaphane suppresses high fat diet-induced hepatic steatosis in mice mediated through the inhibition of inflammasome in liver

**Figure 1:**
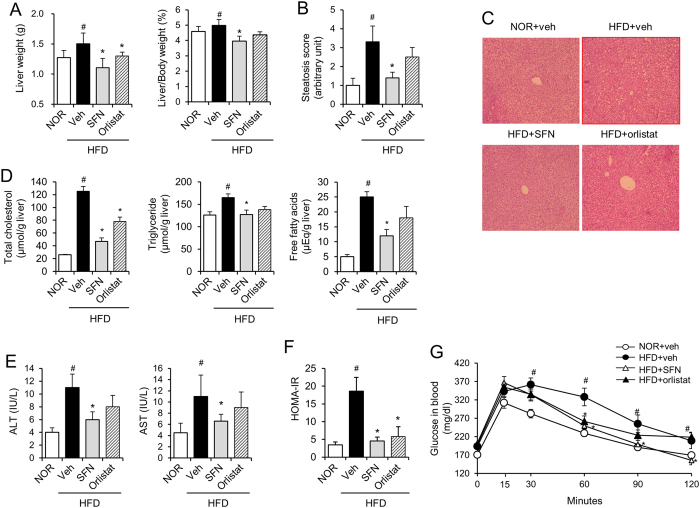
Oral administration of sulforaphane ameliorates high fat diet-induced hepatic steatosis in mice. Mice were fed normal chow diet (NOR) or a high-fat diet (HFD) for 9 weeks. SFN (30 mg/kg/day) or orlistat (10 mg/kg/day) was administered once daily via oral gavage for 9 weeks. N = 8/group. (**A**) Liver weights (left panel), Liver weight/body weight (right panel). (**B**) Hepatic steatosis scores were determined with semi-quantitative pathological standards. (**C**) The liver tissues were stained with hematoxylin and eosin. (**D**) The hepatic levels of total cholesterol, triglycerides, and free fatty acids were determined. (**E**) ALT and AST serum levels were determined. (**F**) Serum homeostasis model assessment of insulin resistance (HOMA-IR) levels in mice. (**G**) For intraperitoneal glucose tolerance testing (IPGTT), glucose solutions (1 g/kg) were administered via intraperitoneal injection to mice after a 12-hr fasting. Blood glucose levels were measured at 0, 15, 30, 60, 90 and 120 min after the glucose challenge. Values are presented as the mean ± SEM (n = 8/group). ^#^Significantly different from NOR alone, *p* < 0.05. *Significantly different from HFD + veh, *p* < 0.05. Veh, vehicle.

We investigated whether oral administration of SFN resulted in prevention of NAFLD associated with high fat intake. Mice were fed a normal chow (NOR) or high-fat diet (HFD) for 9 weeks with or without daily oral administration of SFN (30 mg/kg/day). Orlistat (10 mg/kg/day), an anti-obesity drug, was administered as a positive control. Consumption of high levels of fat resulted in the development of hepatic steatosis in mice, compared with mice fed NOR, as shown by an increase in liver weight, histological steatosis score, serum levels of ALT and AST, and hepatic levels of total cholesterol, triglycerides, and free fatty acids (Fig. 1).

Daily oral administration of SFN for 9 weeks culminated in a reduction in the liver weight and hepatic steatosis scores increased by HFD (NOR + veh, 1.0; HFD + veh, 3.2; HFD + SFN, 1.3; HFD + orlistat, 2.4) (Fig. 1A,B). Consistently, histological examination indicated that lipid deposition in the livers of HFD-fed mice was inhibited by administration of SFN (Fig. 1C). SFN treatment decreased the levels of hepatic total cholesterol, triglycerides, and free fatty acids elevated by HFD (Fig. 1D). Furthermore, SFN treatment resulted in a reduction of the serum levels of ALT and AST increased by HFD (Fig. 1E). The suppressive effects of SFN on hepatic steatosis were comparable to orlistat, an anti-obesity drug. We examined whether suppression of hepatic steatosis by SFN was accompanied by the restoration of an HFD-induced metabolic disorder. The homeostasis model assessment of insulin resistance index (HOMA-IR) and glucose tolerance test indicated that oral administration of SFN prevented the insulin resistance induced by HFD (Fig. 1F,G).

**Figure 2:**
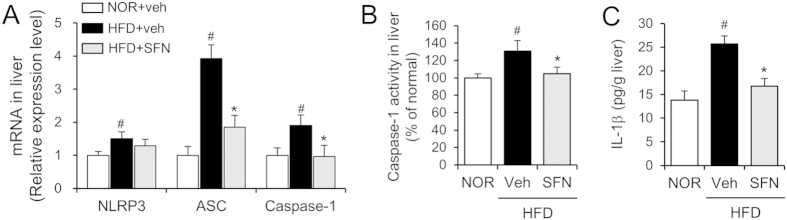
Oral administration of sulforaphane suppresses high fat diet-induced inflammasome activation in mouse livers. Mice were fed normal chow diet (NOR) or a high-fat diet (HFD) for 9 weeks. Sulforaphane (SFN, 30 mg/kg/day) was administered once daily via oral gavage for 9 weeks. N = 8/group. (**A**) NLRP3, ASC and caspase-1 mRNA levels in the liver were determined by quantitative real time PCR. mRNA levels are expressed as the relative expression levels compared to the NOR + veh group for each gene. (**B**) The enzymatic activity of caspase-1 in liver homogenates was determined using a Caspase-1 assay kit and expressed as the relative activity to NOR alone. (**C**) IL-1β protein levels in liver homogenates were determined by ELISA. Values are presented as the mean ± SEM (n = 8/group). ^#^Significantly different from NOR alone, *p* < 0.05. *Significantly different from HFD + veh, *p* < 0.05. Veh, vehicle.

To investigate whether the preventive effect of SFN on hepatic steatosis was associated with the suppression of the NLRP3 inflammasome *in vivo*, we determined the expression levels of NLRP3 inflammasome components (including NLRP3, ASC, and caspase-1) in the livers. ASC and caspase-1 expression was remarkably increased by HFD, whereas NLRP3 expression was slightly enhanced (Fig. 2A). Oral administration of SFN inhibited HFD-induced upregulation of ASC and caspase-1 in the liver (Fig. 2A). We further determined the enzymatic activity of caspase-1 and the protein level of IL-1β as hallmarks of inflammasome activation in the liver isolated from mice fed NOR or HFD for 9 weeks with or without SFN. HFD increased the enzymatic activity of caspase-1 and IL-1β protein levels in the liver, whereas oral administration of SFN reduced the increase of the caspase-1 activity and IL-1β protein level in the liver caused by HFD (Fig. 2B,C). These results demonstrate that oral administration of SFN resulted in the suppression of HFD-induced NLRP3 inflammasome activation in the mouse liver.

These results demonstrate that oral administration of SFN was effective in preventing HFD-induced hepatic steatosis and was beneficial in improving the insulin resistance induced by HFD. This prevention was potentially mediated by suppression of fat-induced NLRP3 inflammasome activation.

### Sulforaphane suppresses the activation of the NLRP3 inflammasome induced by saturated fatty acid in primary hepatocytes

**Figure 3:**
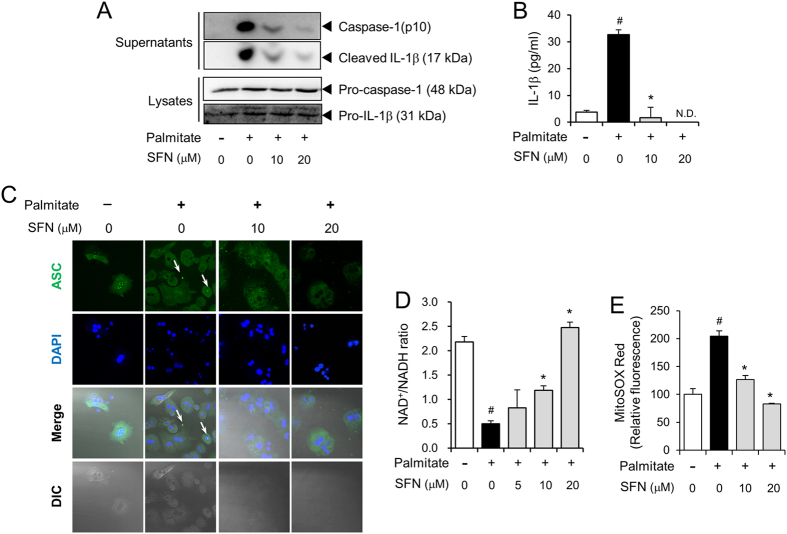
Sulforaphane suppresses activation of the NLRP3 inflammasome induced by saturated fatty acid in primary mouse hepatocytes. After primary mouse hepatocytes were primed with LPS (500 ng/ml) for 4 hr, cells were treated with SFN for 1 hr at the indicated concentrations and further stimulated with palmitate (400 μM) for 16 hr. (**A**) Immunoblotting for caspase-1 (p10), IL-1β, pro-caspase-1, and pro-IL-1β was performed with cell culture supernatants and cell lysates. Cropped blots are presented and the full-length blots are included in the Supplementary Figure 1. The gels have been run under the same experimental conditions. (**B**) IL-1β secretion into the culture medium was determined by ELISA. N.D., not detected. (**C**) Cells were stained for ASC (green) and 4′,6-diamidino-2-phenylindole (DAPI, 1 μg/ml, blue) and assessed by confocal microscopy. Arrows indicate ASC speckles. Data are representative of three independent experiments. DIC, differential interference contrast. (**D**) The NAD^+^/NADH ratio in cell lysates was determined using a commercial kit. (**E**) Cells were stained with MitoSOX Red (4 mM) to detect mitochondrial superoxide and DAPI for nuclei staining and assessed by confocal microscopy. MitoSOX Red fluorescence was presented as relative fluorescence compared with the vehicle alone. (**B**,**D**,**E**) Values are presented as the mean ± SEM. ^#^Significantly different from vehicle alone, *p* < 0.05. *Significantly different from palmitate alone, *p* < 0.05. Veh, vehicle.

We investigated whether SFN inhibited the activation of the NLRP3 inflammasome induced by the saturated fatty acid, palmitate, in primary mouse hepatocytes. Palmitate-induced degradation of pro-caspase-1 to caspase-1(p10) as well as cleavage of pro-IL-1β to mature IL-1β in primary mouse hepatocytes, as determined by immunoblotting of caspase-1(p10) and IL-1β in cell supernatants (Fig. 3A). These results indicate that palmitate induced activation of the NLRP3 inflammasome in hepatocytes. SFN inhibited palmitate-induced production of caspase-1(p10) and mature IL-1β in hepatocyte cell supernatant (Fig. 3A). SFN suppressed palmitate-induced secretion of mature IL-1β into cell culture medium as determined by ELISA (Fig. 3B). Consistently, SFN prevented palmitate-induced oligomerization of ASC, another hallmark of inflammasome activation, as shown by speckle formation using confocal microscopy (Fig. 3C). These results demonstrate that SFN suppressed the activation of the NLRP3 inflammasome induced by saturated fatty acid in hepatocytes.

Mitochondrial dysfunction is the preceding event occurring with saturated fat-induced activation of the NLRP3 inflammasome^13^,^17^,^18^. Therefore, we investigated whether SFN prevented the mitochondrial dysfunction induced by palmitate in primary mouse hepatocytes. The ratio of oxidized to reduced nicotinamide adenine dinucleotide (NAD^+^/NADH) was decreased after treatment of palmitate due to mitochondrial dysfunction, while the NAD^+^/NADH ratio was recovered by SFN treatment (Fig. 3D). We next determined mitochondrial reactive oxygen species (ROS) content that can be increased by mitochondrial dysfunction and is crucial in inflammasome activation^19^. SFN reduced palmitate-induced production of mitochondrial ROS in hepatocytes (Fig. 3E). These results show that SFN prevented the mitochondrial dysfunction induced by saturated fatty acid, thereby suppressing NLRP3 inflammasome activation in hepatocytes.

### Sulforaphane induces activation of the AMPK-autophagy axis in mouse primary hepatocytes

**Figure 4:**
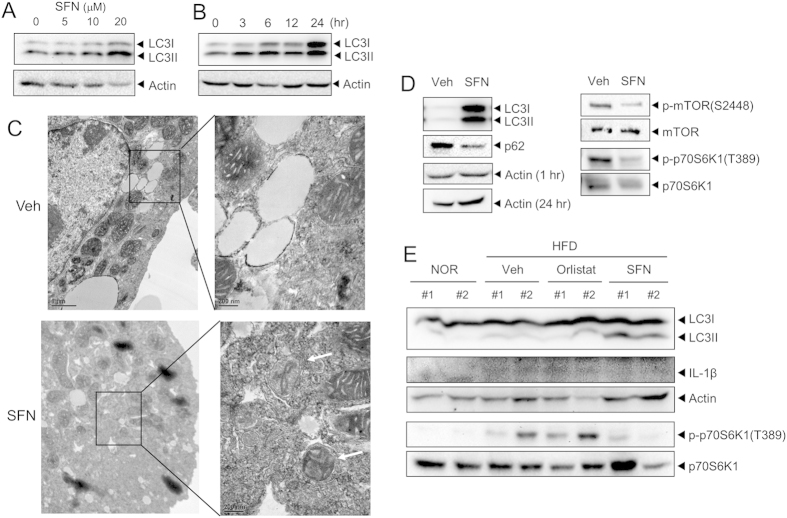
Sulforaphane induces autophagy by the suppression of mTOR in hepatocytes. (**A,B**) Primary mouse hepatocytes were treated with SFN as indicated (24 hr for (**A**); 20 μM for (**B**)). Cell lysates were analyzed with immunoblot analysis for LC3I, LC3II, and actin. (**C**) After primary mouse hepatocytes were treated with SFN (20 μM) for 24 hr, cells were assessed by transmission electron microscopy analysis (original magnification, 8000×). White arrows indicate the autophagosomes/autolysosomal structures. (**D**) After primary mouse hepatocytes were treated with SFN (20 μM) for 1 hr (except LC3 for 24 hr), cell lysates were analyzed by immunoblotting for LC3, p62, actin, phospho-mTOR(S2448), mTOR, phospho-p70S6K1(T389), and p70S6K1. (**E**) Mice were fed a normal chow (NOR) or a high-fat diet (HFD) for 9 weeks with or without daily oral administration of SFN (30 mg/kg/day) or orlistat (10 mg/kg/day) once per day for 9 weeks. Liver samples were analyzed by immunoblotting for LC3, IL-1β, actin, phospho-p70S6K1(T389) and p70S6K1. #1 and #2 represent independent liver samples. Veh, vehicle. (**A**,**B**,**D**,**E**) Cropped blots are presented and the full-length blots are included in the Supplementary Figures 2–5. The gels have been run under the same experimental conditions.

Given that autophagy is linked to quality control of mitochondria by removing damaged or dysfunctional mitochondria, defective autophagy events lead to the accumulation of dysfunctional mitochondria, subsequently stimulating the activation of the NLRP3 inflammasome^17^,^20^. Recent studies have demonstrated essential protective function for autophagy during liver injury and death by inhibiting the generation of inflammasome-depenent IL-1β^21^. Immunoblotting results of LC3I conversion to LC3II indicated that SFN induced autophagosome formation in primary mouse hepatocytes (Fig. 4A,B). Transition electron microscopic analysis confirmed that autophagosome formation was induced by SFN in primary mouse hepatocytes (Fig. 4C). Activation of autophagy by SFN resulted in p62 degradation (Fig. 4D); p62 is an autophagy substrate that is degraded during autophagy activation. These suggest that SFN promotes autophagy. Mammalian target of rapamycin (mTOR) is a negatively regulator of autophagy. SFN reduced the phosphorylation of mTOR(Ser2448) in primary mouse hepatocytes (Fig. 4D), suggesting that SFN inhibited mTOR activation. SFN suppression of mTOR activation was confirmed by a decrease in p70S6K1 phosphorylation, which is a downstream substrate of mTOR (Fig. 4D).

To investigate whether SFN regulated fat-mediated decrease in autophagy, the effect of SFN on autophagy was examined in livers isolated from mice fed NOR, HFD, or HFD with SFN. Oral administration of SFN induced LC3II formation in the liver (Fig. 4E). Autophagy induction by SFN was correlated with the suppression of IL-1β generation (Fig. 4E). The suppression of mTOR in livers by SFN was confirmed by the reduction in the phosphorylation of p70S6K1, which was increased by HFD (Fig. 4E). The results show that oral administration of SFN prevented fat-induced impairment of autophagy in mouse liver. These results further demonstrate the regulation of autophagy-inflammasome axis by SFN both *in vitro* and *in vivo*.

### 

**Figure 5:**
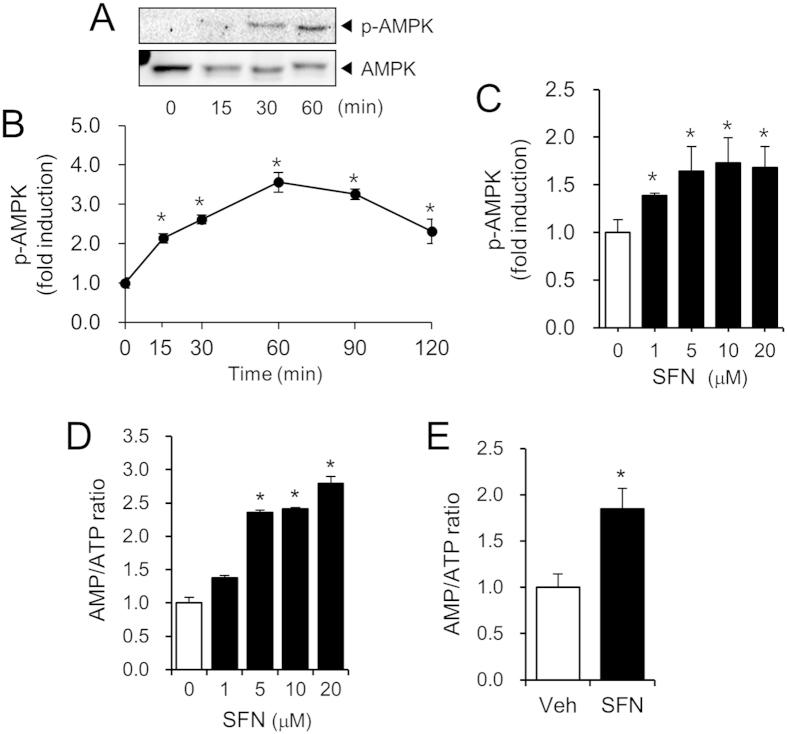
Sulforaphane induces activation of AMPK in hepatocytes. (**A**,**B**) Primary mouse hepatocytes were treated with SFN (20 μM) for the indicated times. For (**A**), cell lysates were analyzed by immunoblotting for phospho-AMPK(Thr172) and AMPK. Cropped blots are presented and the full-length blots are included in the Supplementary Figure 6. The gels have been run under the same experimental conditions. For (**B**), the levels of phospho-AMPK(Thr172) were determined by ELISA kit and expressed as fold inductions compared with time 0. (**C**) After primary mouse hepatocytes were treated with SFN at the indicated concentrations for 1 hr, phospho-AMPK(Thr172) levels were determined by ELISA and expressed as fold inductions compared with the vehicle alone. (**D**) After primary mouse hepatocytes were treated with SFN at the indicated concentrations for 24 hr, AMP and ATP levels were determined. The AMP/ATP ratio was expressed relative to the vehicle alone. (**E**) After mice were orally administered SFN (20 mg/kg/day) for 5 days, AMP and ATP levels in the liver were determined. The AMP/ATP ratio was expressed relative to the vehicle alone. (**B**–**E**) Values are presented as the mean ± SEM. *Significantly different from the vehicle alone, *p* < 0.05.

Activation of AMP-activated protein kinase (AMPK) by saturated fatty acid is involved in the regulation of autophagy events. Therefore, we investigated whether SFN induced the activation of AMPK in hepatocytes. SFN induced phosphorylation of threonine 172 in the AMPK-α subunit in primary mouse hepatocytes in a time- and dose-dependent manner (Fig. 5A,B,C), suggesting that SFN activated AMPK in hepatocytes. SFN increased the AMP/ATP ratio in hepatocytes (Fig. 5D), suggesting that an increase in AMP and a decrease in ATP resulted in the SFN-induced activation of AMPK. To confirm the effect of SFN on hepatic energy changes *in vivo*, SFN (20 mg/kg) was orally administered to mice for 5 days, and the levels of adenine nucleotides were examined in mouse livers. Oral administration of SFN resulted in a decrease in hepatic ATP levels and an increase in hepatic AMP levels, with a 1.8-fold increase in the AMP/ATP ratio compared with vehicle treatment (Fig. 5E). These results indicate that SFN increased the ratio of AMP to ATP in hepatocytes, culminating in activation of AMPK both *in vitro* and *in vivo*.

**Figure 6:**
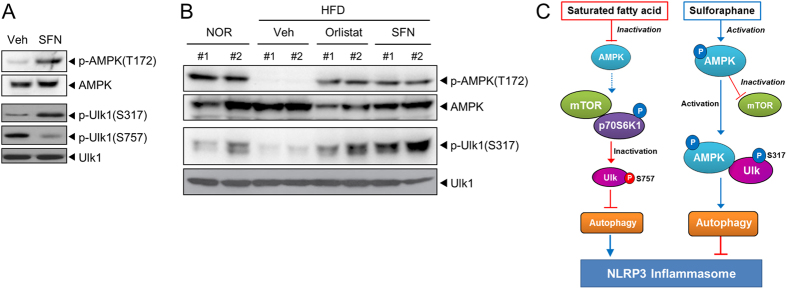
Activation of AMPK by sulforaphane is associated with the induction of autophagy in primary mouse hepatocytes and mouse livers from high-fat diet-treated mice. (**A**) After primary mouse hepatocytes were treated with SFN (20 μM) for 1 hr, cell lysates were analyzed by immunoblotting for phospho-AMPK(T172), AMPK, phospho-Ulk1(S317), phospho-Ulk1(S757), and Ulk1. (**B**) Mice were fed a normal chow (NOR) or a high-fat diet (HFD) for 9 weeks with or without daily oral administration of SFN (30 mg/kg/day) or orlistat (10 mg/kg/day) once per day for 9 weeks. Liver samples were analyzed by immunoblotting for phospho-AMPK(T172), AMPK, phospho-Ulk1(S317), and Ulk1. #1 and #2 represent independent liver samples. Veh, vehicle. (A, B) Cropped blots are presented and the full-length blots are included in the Supplementary Figures 7 and 8. The gels have been run under the same experimental conditions. (**C**) Differential regulation of signaling pathways involving AMPK, mTOR, p70S6K1, Ulk1, and autophagy by saturated fatty acid (palmitate) and SFN is depicted.

To further confirm SFN activated the AMPK-autophagy axis in hepatocytes, we examined the regulation of the signaling pathways from AMPK to autophagy by SFN. AMPK acts as a positive regulator of autophagy by directly phosphorylating the mammalian autophagy-initiating kinase Ulk1, a homologue of yeast Autophagy related-1 (ATG1), at Ser317. SFN induced the phosphorylation of Ulk1 at Ser317 in primary mouse hepatocytes (Fig. 6A). In contrast, the phosphorylation of Ulk1 at Ser757 was decreased by SFN (Fig. 6A). These suggest that SFN promotes autophagy by activating AMPK-Ulk1 pathway.

To investigate whether SFN regulated fat-mediated decrease in autophagy, the effect of SFN on signaling pathways from AMPK to autophagy was examined in the liver isolated from mice fed NOR, HFD, or HFD with SFN. HFD decreased the phosphorylation of AMPK(T172) and Ulk(S317) in the liver, whereas oral administration of SFN to mice reversed the HFD-induced downregulation of p-AMPK(T172) and p-Ulk1(S317) in liver tissues (Fig. 6B). These results demonstrate the activation of AMPK-autophagy pathway by SFN both *in vitro* and *in vivo*. In addition, the results show that oral administration of SFN prevented fat-induced impairment of autophagy in mice via the activation of AMPK, followed by Ulk1 activation.

Collectively, our results demonstrate that SFN prevents HFD-induced hepatic steatosis mediated by the suppression of NLRP3 inflammasome activation in the liver. The activation of AMPK and autophagy by SFN leads to a recovery from the mitochondrial dysfunction induced by saturated fatty acids, thereby downregulating fat-induced activation of the NLRP3 inflammasome (Fig. 6C).

## Discussion

These results suggest that regulating NLRP3 inflammasome activation with small molecule inhibitors is a potential therapeutic strategy to treat or prevent the pathological outcomes of NAFLD. Csak *et al.*^12^ suggested that an increased influx of saturated fatty acids to the liver leads to activation of the NLRP3 inflammasome followed by IL-1β secretion and a subsequent inflammatory response. IL-1β is an important pro-inflammatory cytokine that amplifies inflammation and sensitizes hepatocytes to liver damage^10^. We identified SFN as a potent inhibitor of the NLRP3 inflammasome activation induced by a HFD or saturated fatty acid, as shown by a decrease in IL-1β and caspase-1 production in the liver. Our results demonstrate the inhibitory effects of SFN on NLRP3 inflammasome activation both *in vitro* in hepatocytes and an *in vivo* in an animal model.

Fatty acid-induced inflammasome activation in macrophages was NLRP3 dependent and involved increased mitochondrial production of reactive oxygen species (ROS) and decreased autophagy due to reduced AMPK activity^13^. A previous paper reported that metformin is a known AMPK activator that inhibits IL-1β production by macrophages of diabetic patients through AMPK activation *in vitro*^22^. Our results demonstrate that SFN activates AMPK and autophagy, whereas it suppresses the activation of the NLRP3 inflammasome and mitochondrial impairment in hepatocytes. These results are consistent with the hypothesis that AMPK activation attenuates inflammasome activation, which might involve rejuvenation of ‘stressed’ mitochondria, as dysfunctional mitochondria accelerate NLRP3 inflammasome activation^23^.

NAFLD is prevalent in women as well as men. Some studies showed that women had a higher prevalence as compared with men. Ayonrinde *et al.* reported in the Western Australian Pregnancy Cohort Study that women compared with men had a significantly higher prevalence of NAFLD and central obesity^24^. Interestingly, men with NAFLD showed more adverse metabolic phenotype and more severe liver injury than women with NAFLD^24^. In contrast, other population-based studies showed that NAFLD is significantly more prevalent in men than in women^25^. However, Younossi *et al.* reported that lean NAFLD was associated more with women^26^. These underscore the complexity of the influence of gender on the prevalence, severity, and phenotype of NAFLD. There are several factors suggested to contribute to the gender difference of NAFLD, including sex hormones, alcohol consumption, lifestyle, and environment. It is postulated that female hormones may protect against NAFLD since NAFLD is twice as common in postmenopausal women as in premenopausal women^27^,^28^. In our study, we chose male mice to exclude the influence of the female sex hormone cycle on the experiment because we intended to make the experimental system more consistent during 9-week diet study. It is important to compare any gender difference in SFN response because this would be the next step to advance towards the clinical application of our findings. It would be pursued in a future study to investigate whether there are gender-specific differences in the protective effect of SFN in NAFLD.

Collectively, our results demonstrate that SFN prevents HFD-induced hepatic steatosis mediated by the suppression of NLRP3 inflammasome activation in hepatocytes. The induction of autophagy by SFN leads to a recovery from the mitochondrial dysfunction induced by saturated fatty acid, thereby downregulating fat-induced activation of the NLRP3 inflammasome through AMPK-autophagy signaling. The subsequent decrease in IL-1β production in hepatocytes contributes to alleviating hepatic steatosis. These results suggest that modulation of NLRP3 inflammasome with small molecule inhibitors is a potentially effective strategy to prevent the pathological outcomes of NAFLD.

## Materials and Methods

### Ethics statement

All animals received humane care according to the criteria outlined in the “Guide for the Care and Use of Laboratory Animals” prepared by the National Academy of Sciences and published by the National Institutes of Health (NIH publication 86–23 revised 1985). All the experimental procedures were carried out in accordance with the protocols approved by the Institutional Animal Care and Use Committee (IACUC) of the Catholic University of Korea (permission# 2014-015).

### Animals

C57BL/6 mice (male) were purchased from Orient bio (Seoul, Korea) and were acclimated under specific pathogen-free conditions in an animal facility for at least a week before experimentation. The mice were housed in a 12:12-hr light/dark cycle, temperature (23 ± 3 °C) and humidity (40–60%) controlled room. All mice were allowed *ad libitum* access to their diet and water during the experimental period.

### Reagents

Purified lipopolysaccharides (LPS) from *Escherichia coli* were obtained from List Biological Laboratory (Campbell, CA) and dissolved in endotoxin-free water. SFN, orlistat, and palmitate were purchased from Sigma-Aldrich (St. Louis, MO). Antibodies against actin, caspase-1, p-Ulk1(S317), p-Ulk1(S757), and Ulk1were obtained from Santa Cruz Biotechnology, Inc. (Dallas, TX). An antibody against IL-1β was obtained from R&D Systems (Minneapolis, MN). Antibodies against AMPK, phospho-AMPK(T172), mTOR, phospho-mTOR(S2488), S6K1, phospho-p70S6K1(T389), LC3І, LC3II, and p62 were obtained from Cell Signaling Technology (Danvers, MA).

### Preparation of primary mouse hepatocytes

Primary mouse hepatocytes were obtained by collagenase perfusion of livers as described previously^29^, with slight modifications. Mouse livers were perfused with phosphate buffered saline (PBS) containing type IV collagenase (100 U/ml; Worthington Biochemical, Lakewood, NJ) for digestion at a flow rate of 9 ml/min. Hepatocytes were isolated in Dulbecco’s Modified Eagle Medium (DMEM) supplemented with 25 mM glucose and 10% fetal bovine serum (FBS). Viability was >90% for all preparations, as determined by trypan blue staining. Prior to experimentation, cells were plated on collagen-coated (8 μg/cm^2^) plates, maintained overnight in serum-free DMEM and used within 30 hr of plating.

### High-fat diet study

C57BL/6 mice (males, 5 weeks-old) were randomly divided into four weight-matched groups (n = 8/group): normal chow diet (NOR), high-fat diet (HFD), HFD + SFN and HFD + orlistat. Mice were fed a HFD for 9 weeks, accompanied by daily oral administrations of either SFN (30 mg/kg) or orlistat (10 mg/kg). NOR (11.5 kcal%) and HFD (40 kcal%) were obtained from Research Diets, Inc. (New Brunswick, NJ, USA). Body weight and food intake were recorded every week. At the end of the 9-week period, all animals were fasted for 12 hr. On the following day, mice were anesthetized with Zoletil (Virbac, Carros Cedex, France), and blood samples were collected by cardiac puncture. Liver tissues were excised, rinsed, weighed, and stored at −70 °C pending further analysis.

### Immunoblot assay with hepatocytes and liver tissue

For immunoblotting of caspase-1 and IL-1β, hepatocytes were plated in 6-well plates (8 × 10^5^ cells/ml) and primed with LPS (500 ng/ml) for 4 hr. Cells were washed with PBS to remove LPS before exposure to SFN to exclude any effect of SFN on LPS. Cells were treated with SFN for 1 hr and further stimulated with palmitate in serum-free medium. To detect caspase-1(p10) and IL-1β in cell culture supernatant, the supernatants were precipitated by adding one volume of methanol and 0.25 volumes of chloroform followed by centrifugation at 20,000 g for 10 min. The upper phase was discarded, and one volume of methanol was added. The mixture was centrifuged at 20,000 g for 10 min to obtain a protein pellet, which was dried at room temperature, resuspended in Laemmli buffer (0.25 M Tris-HCl, pH 6.8, 0.4% glycerol, 10% SDS, 0.2% 2-mercaptoethanol and 0.64% bromophenol blue) and subjected to sodium dodecyl sulfate (SDS)-polyacrylamide gel electrophoresis (PAGE). To detect pro-caspase-1 and pro-IL-1β, cell pellets were lysed in RIPA buffer (50 mM Tris-HCl, pH 7.4, 1% NP-40, 0.25% sodium deoxycholate, 150 mM NaCl, 1 mM EGTA, 1 mM PMSF, 1 mM Na_3_VO_4_, 10 μg/ml aprotinin, and 10 μg/ml leupeptin) and processed for SDS-PAGE. Liver tissues isolated from experimental mice were homogenized at 4 °C in RIPA buffer and centrifuged at 10,000 g for 10 min at 4 °C; protein extracts processed for SDS-PAGE. After electrophoresis, proteins were transferred to polyvinylidene difluoride membranes (Millipore, Billerica, MA). The membranes were probed with primary antibodies and their corresponding horseradish peroxidase (HRP)-conjugated secondary antibodies. The bands were visualized using an enhanced chemiluminescence (ECL) system.

### Enzyme-linked immunosorbent assays

IL-1β levels in culture media or liver homogenate supernatants were determined using a DuoSet enzyme-linked immunosorbent assay (ELISA) kit (R&D systems) according to the manufacturer’s instructions. Standard curve concentrations were 15.6 to 1000 pg/ml.

### Confocal microscopy analysis

Primary hepatocytes were plated overnight on cover slides in 24-well plates. After cells were fixed with methanol for 30 min, cells were blocked with 1% bovine serum albumin for 1 hr and incubated with an anti-ASC antibody (Santa Cruz Biotechnology Inc.) at 4 °C overnight. Cells were further incubated with Alexa Fluor 488 goat anti-rabbit antibody (Invitrogen, Carlsbad, CA) for 2 hr at room temperature. To determine mitochondrial superoxide production, hepatocytes were stained with MitoSOX Red (4 mM; Invitrogen). Cells were co-stained with 4′,6-diamidino-2-phenylindole (DAPI, 1 μg/ml; Invitrogen) for nuclei staining. Slides were mounted in fluorescent mounting medium (Vector laboratories, Burlingame, CA). Samples were examined with an LSM710 laser scanning confocal microscope (Carl Zeiss, Oberkochen, Germany). Images were obtained and analyzed with ZEN2011 software (Carl Zeiss).

### Measurement of the NAD^+^/NADH ratio

Quantification of NAD and NADH was performed using a NAD^+^/NADH assay kit from Abcam (Cambridge, UK) according to the manufacturer’s instructions. Livers were snap frozen in liquid nitrogen and extracted at 4 °C using the extraction buffer provided with the NAD^+^/NADH assay kit. NAD^+^ and NADH values were normalized by their protein concentration.

### Reverse transcription and quantitative real-time polymerase chain reaction (qRT-PCR) analysis

Total RNAs from mouse livers or cultured cells were extracted using Trizol reagent (Invitrogen) according to the manufacturer’s instructions. Reverse transcription and qRT-PCR analysis was performed as described previously^30^. Primers used were as follows: *caspase-1*, 5′-AGATGGCACATTTCCAGGAC-3′ and 5′-GATCCTCCAGCAGCAACTTC-3′; *nlrp3*, 5′-AGCCTTCCAGGATCCTCTTC-3′ and 5′-CTTGGGCAGCAGTTTCTTTC-3′; *asc*, 5′-GAAGCTGCTGACAGTGCAAC-3′ and 5′-GCCACAGCTCCAGACTCTTC-3′; *actin*, 5′-TCATGAAGTGTGACGTTGACATCCGT-3′ and 5′-TTGCGGTGCACGATGGAGGGGCCGGA-3′. Specificity of the amplified PCR products was assessed by melting curve analysis, and the gene expression was normalized to the corresponding actin level.

### Caspase-1 activity assay

The enzymatic activity of caspase-1 in mouse livers was measured using a Caspase-1 assay kit from Bio-vision (Milpitas, CA) according to the manufacturer’s instructions. Fluorescence was recorded at 400 nm after excitation at 505 nm using a SpectraMaxM5 microplate reader (Molecular Devices, Sunnyvale, CA).

### Histological analysis

Liver tissue specimens were fixed in 10% buffered formalin, embedded in paraffin, cut at 5-μm thickness and stained with hematoxylin and eosin for histological examination of fat droplets. Steatosis was numerically scored following semi-quantitative pathological standards^31^.

### Biochemical analysis

The activities of alanine aminotransferase (ALT) and aspartate aminotransferase (AST) in serum were determined with an Express Plus Biochemistry analyzer (Chiron Diagnostics, Emeryville, CA). Hepatic lipids were extracted as described by Folch *et al.*^32^ using a chloroform-methanol mixture (2:1 v/v), and the dried lipid residues were dissolved in 2 ml ethanol. The concentrations of cholesterol, triglycerides, and free fatty acids in the hepatic lipid extracts were measured using commercial kits (Bio-Clinical System, Korea). Blood insulin levels were assayed using ELISA kits (American Laboratory Products Company, Salem, NH). Blood glucose levels were determined by a Glucocard X-Meter (Arkray, Kyoto, Japan). To perform an intraperitoneal glucose tolerance test (IPGTT), glucose solutions (2 g/kg) were administered via intraperitoneal injection to mice after 12-hr fasting. Blood glucose levels were measured at 0, 15, 30, 60, 90, and 120 min after the glucose challenge.

### Determination of AMP-activated protein kinase activity

Activity of AMP-activated protein kinase (AMPK) was determined by measuring phosphorylation of AMPK at Thr172 using an ELISA kit (Invitrogen) according to the manufacturer’s instructions. The analytical sensitivity was <1 unit/ml of AMPKα(pT172).

### ATP/AMP Assay

Intracellular ATP and AMP concentrations were determined with the ATP/ADP/AMP Assay kit (Biomedical Research Service & Clinical Application, Buffalo, NY). In the presence of ATP, the enzyme luciferase catalyzes the oxidation of luciferin with concomitant emission of yellow green light. Measurements were made on a luminometer (Berthold technologies, Bad Wildbad, Germany) and compared with a standard curve of ATP concentrations. AMP concentration was calculated according to the manufacturer’s instruction.

### Transmission electron microscopy (TEM) analysis

Primary mouse hepatocytes were seeded onto four-chambered coverglass (Lab-Tek Chambered Coverglass System; Nalgene-Nunc, Rochester, NY) at a density of 2 × 10^4^ cells/ml. Cells were fixed with 2.5% glutaraldehyde in 0.1 M sodium cacodylate and 1 mM CaCl_2_ (pH 7.4) and postfixed in 1% osmium tetroxide (OsO_4_). Then, the samples were dehydrated in graded ethanol, from 50% to 100%, and embedded in Spurr’s resin. Ultrathin sections (90 nm) were cut and placed on 150 mesh copper grids. After staining with lead citrate and uranyl acetate (2% in 50% ethanol), images were acquired using an Olympus EM208S transmission electron microscope (Olympus, Tokyo, Japan).

### Statistical analysis

Results are expressed as the mean ± standard error of the mean (SEM). One-way analysis of variance was performed followed by the Duncan’s multiple range test to determine any significant differences between groups. SPSS 12.0 software (IBM Co., Chicago, IL) was used. Statistical significance was considered as p < 0.05.

## Additional Information

**How to cite this article**: Yang, G. *et al.* A pharmacological inhibitor of NLRP3 inflammasome prevents non-alcoholic fatty liver disease in a mouse model induced by high fat diet. *Sci. Rep.* **6**, 24399; doi: 10.1038/srep24399 (2016).

## Supplementary Material

Supplementary Information
