# Erratum: A pharmacological inhibitor of NLRP3 inflammasome prevents non-alcoholic fatty liver disease in a mouse model induced by high fat diet

**DOI:** 10.1038/srep26218

**Published:** 2016-05-20

**Authors:** Gabsik Yang, Hye Eun Lee, Joo Young Lee

Scientific Reports 6: Article number: 24399; 10.1038/srep24399 published online: 04142016; updated: 05202016.

This Article contains errors in the Methods section under subheading ‘High-fat diet study’.

“On the following day, mice were anesthetized with Zoletil (Virbac, Carros Cedex, France), and blood samples were collected by cardiac puncture.”

should read:

“On the following day, mice were sacrificed with CO_2_, and immediately, blood samples were collected by cardiac puncture.”

