# Supplementary material for: A pharmacological inhibitor of NLRP3 inflammasome prevents non-alcoholic fatty liver disease in a mouse model induced by high fat diet
Source: Sci Rep. 2016 Apr 14;6:24399. doi: 10.1038/srep24399 (PMC4830938; doi:10.1038/srep24399)
Supplement: Supplementary Information [file srep24399-s1.pdf]

Supplementary Materials

A pharmacological inhibitor of NLRP3 inflammasome prevents non-alcoholic fatty liver disease in a mouse model induced by high fat diet

Gabsik Yang[1*], Hye Eun Lee[1*], and Joo Young Lee[1‡]

[1]Integrated Research Institute of Pharmaceutical Sciences, College of Pharmacy, The Catholic University of Korea, Bucheon, Republic of Korea, 420-743.

*Gabsik Yang and Hye Eun Lee equally contributed.

Contact Information: ‡Corresponding author: Joo Young Lee, College of Pharmacy, The Catholic University of Korea, Bucheon, Korea, 420-743. Tel: 82-2-2164-4095. Fax: 82-2-2164-4059. E-mail: joolee@catholic.ac.kr.

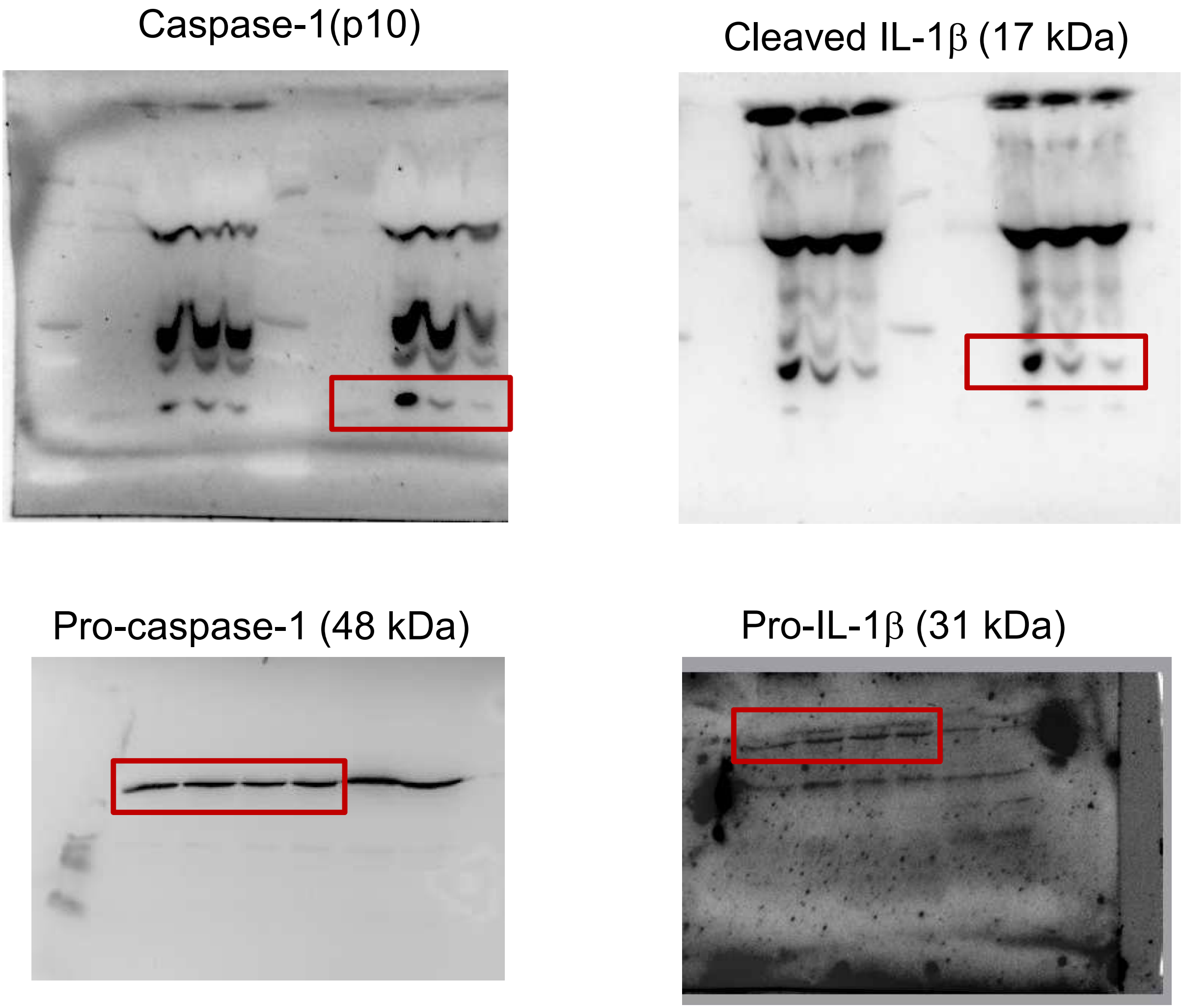

Supplementary Figure 1. Rectangles indicate the cropped blots presented in Figure 3.

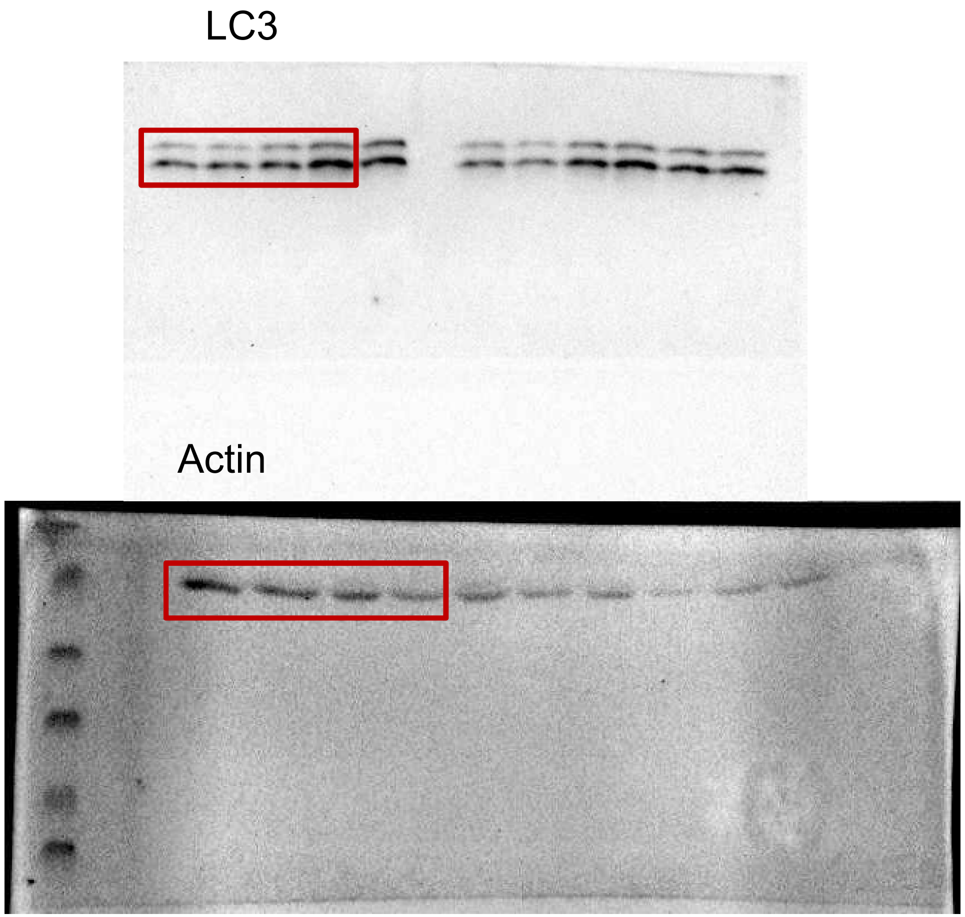

Supplementary Figure 2. Rectangles indicate the cropped blots presented in Figure 4A.

LC3 Actin

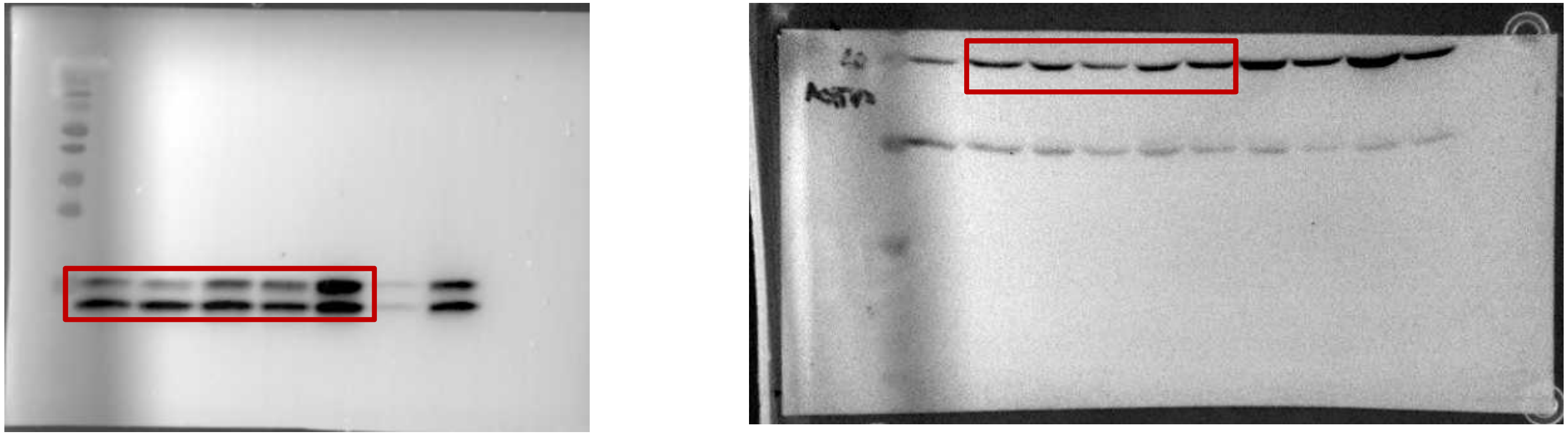

Supplementary Figure 3. Rectangles indicate the cropped blots presented in Figure 4B.

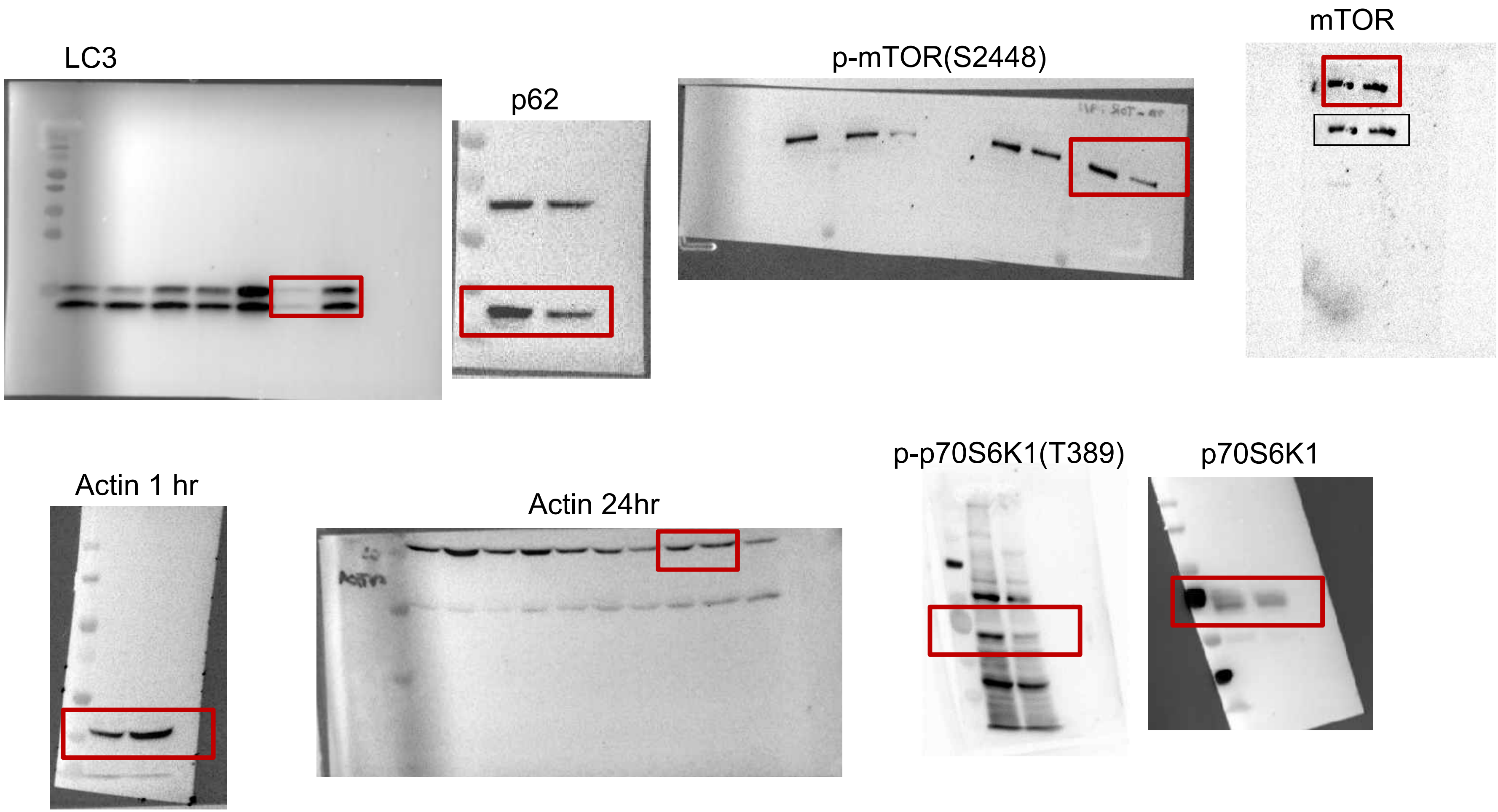

Supplementary Figure 4. Rectangles indicate the cropped blots presented in Figure 4D.

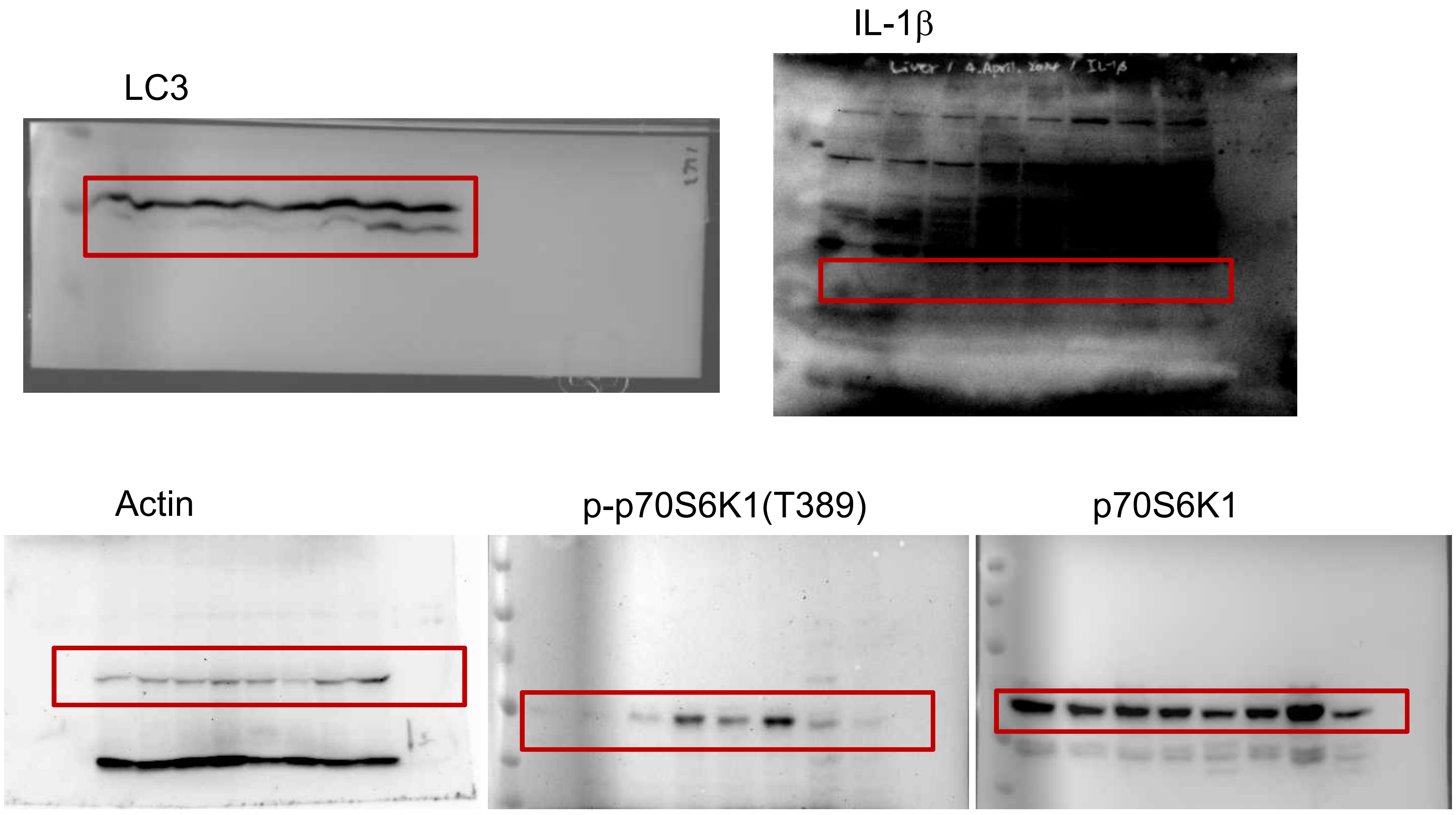

Supplementary Figure 5. Rectangles indicate the cropped blots presented in Figure 4E.

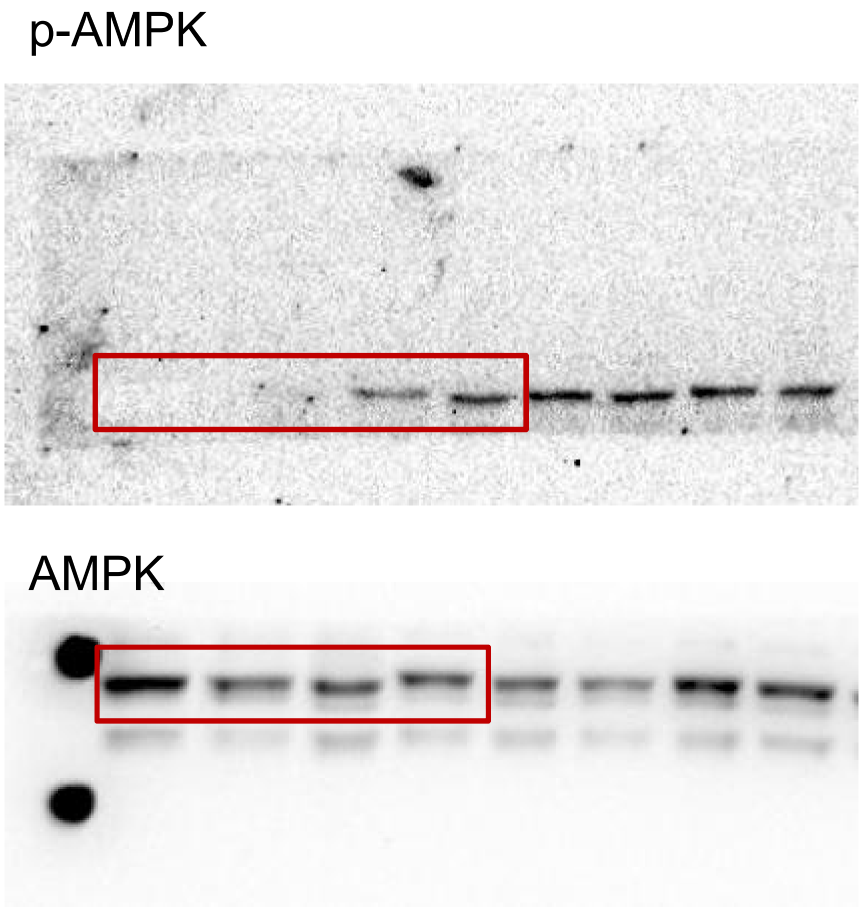

Supplementary Figure 6. Rectangles indicate the cropped blots presented in Figure 5.

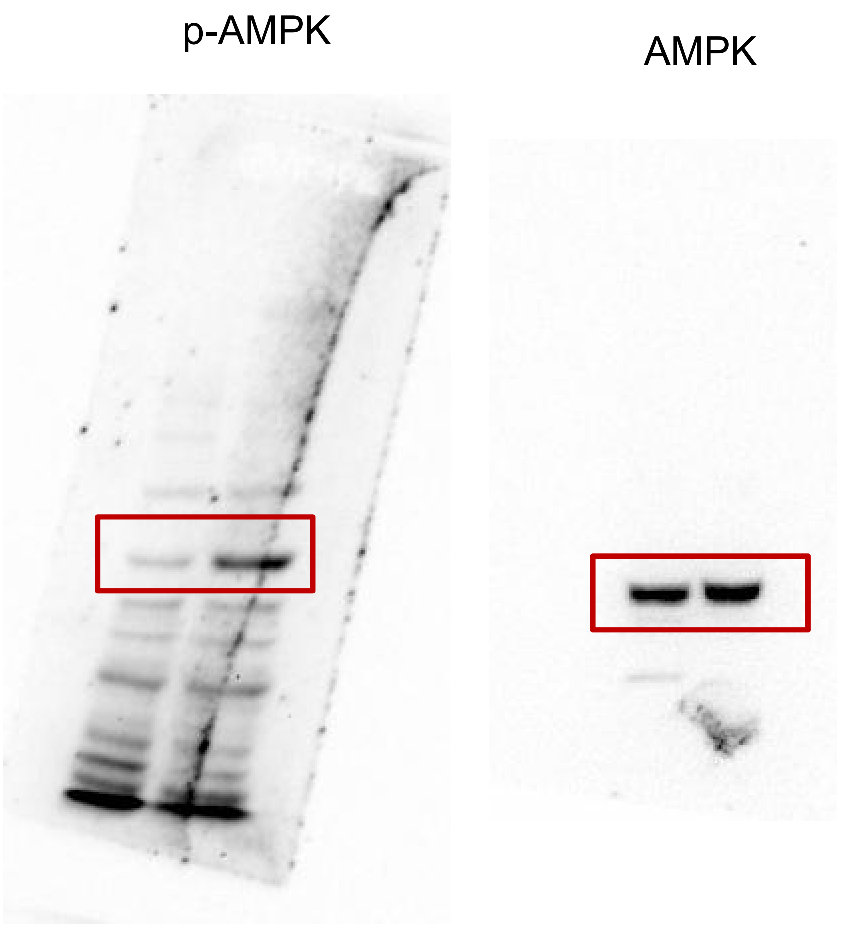

Supplementary Figure 7. Rectangles indicate the cropped blots presented in Figure 6A.

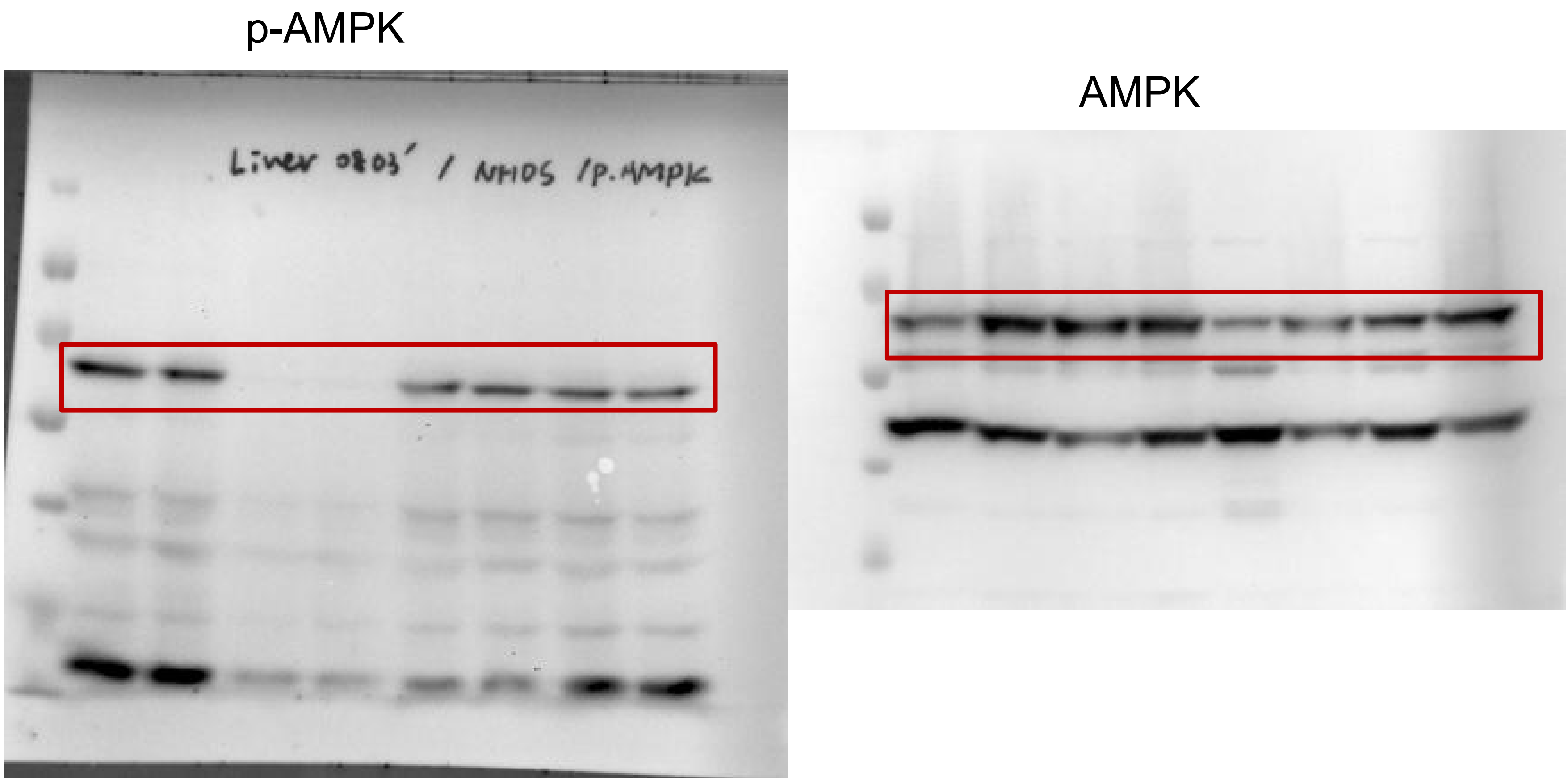

Supplementary Figure 8. Rectangles indicate the cropped blots presented in Figure 6B.
